# Identifying competencies of primary health care teams in Bhavnagar district, Gujarat

**DOI:** 10.1371/journal.pgph.0006861

**Published:** 2026-07-27

**Authors:** Akriti Mehta, Madhavi Misra, Jaclyn Dyson, Tapasvi Puwar, Krishna D. Rao

**Affiliations:** 1 International Health Department, Johns Hopkins Bloomberg School of Public Health, Baltimore, United States of America; 2 Johns Hopkins India Private Limited, New Delhi, India; 3 Indian Institute of Public Health, Gandhinagar, India; Institute of Public Health Bengaluru, INDIA

## Abstract

Primary healthcare (PHC) services provided by multi-disciplinary teams is essential for delivering comprehensive, integrated health services. PHC policies in many low- and middle-income countries emphasize team-based approaches. While competencies (knowledge, skills, and attitudes) of specific health worker cadres have been identified, collective competencies of PHC teams have not been examined. Knowing these competencies will inform development of capacity building initiatives to strengthen PHC team functioning and performance. This study presents findings from an exercise to identify key competencies of PHC teams in Bhavnagar district, Gujarat, India. Team competencies were derived through a multi-step process. We searched the literature to develop an initial list of PHC team functions. We held consultations with technical experts and PHC team members where participants reviewed, edited, and developed a list of PHC team functions and identified corresponding knowledge, skills, and attitudes the PHC team must possess. Participant responses were analyzed and PHC team competencies clustered thematically. PHC team functions comprised taskwork and teamwork activities at community and facility levels. PHC team members viewed their functions through a cadre-specific lens. Identified team competencies were clustered under six themes: technical and clinical, which included use of data and digital tools and ability to implement clinical and technical protocols; communication; shared leadership and accountability; problem-solving; conflict management; and team values. While recognizing the importance of technical and clinical competencies, participants prioritized the “softer” competencies. Findings of this study suggest the importance of training PHC team members on competencies beyond the technical and clinical competencies, that strengthen ways in which teams work together. Further, teams should be trained as a unit to reinforce these competencies. The study also suggests a need for policy and organizational reforms to support team-based care. Strengthening PHC team competencies can enable the team to perform its functions better, contributing to improved service delivery.

## Introduction

Multidisciplinary teams working as a unit are widely recognized as key to providing integrated, comprehensive primary healthcare (PHC) to communities. Global health agencies like the World Health Organization (WHO), and national policies of many countries, emphasize the importance of PHC teams to deliver effective health services [1]. Team-based care involves the “provision of health services to individuals, families, and/or their communities by at least two health providers who work collaboratively with patients and their caregivers – to the extent preferred by each patient – to accomplish shared goals within and across settings to achieve coordinated, high-quality care” [2]. Evidence shows that PHC delivered by a multidisciplinary team is associated with effective, high-quality care [3–5]. Moreover, managing the health of aging populations, addressing the growing burden of non-communicable diseases (NCD), and responding to a wide range of community health needs requires a diverse skill mix among PHC workers [1].

Some of the exemplar PHC models - Brazil’s Family Health Team and Costa Rica’s Basic Integrated Health Care Team (EBAIS) are based on multi-disciplinary teams of PHC workers delivering a range of services to communities. In both settings, PHC teams act as well-coordinated units to deliver essential public health and personal primary care services to a defined set of households [6,7]. In Brazil, the Family Health Team has been associated with greater utilization of primary care, higher vaccination rates, and lower post-neonatal infant mortality rates [6,8]. In Costa Rica, PHC reforms incorporating the EBAIS teams are associated with greater utilization and access to PHC and reductions in infant and adult mortality rates [7,9]. In both countries, delivery of team-based care was supported by the use of technology, appropriate funding and payment systems, and regulatory frameworks [6–9].

### PHC teams in India

In 2018, India undertook important reform efforts to move from selective to comprehensive PHC. A pivotal principle of the reform is to establish a “culture of a team-based approach to the delivery of quality health care encompassing preventive, promotive, curative, rehabilitative and palliative care” [10]. PHC teams at public sector health facilities include multiple health workers, e.g., generally at sub-health centers or Ayushman Arogya Mandirs (AAM; formerly Health and Wellness Centers) the team comprises a Community Health Officer, who is a nurse or an Ayurveda doctor, auxiliary nurse-midwife, and community health workers [10–12]. India’s policy guidelines clearly delineate the PHC team composition at AAMs, along with a comprehensive list of services the team is envisaged to deliver. While guidelines and training documents identify roles, skills, and performance benchmarks of the PHC workforce, efforts have largely been cadre-specific and/or program-specific, with few identifying explicit competencies for the PHC team [13–15].

### Health worker competencies

Competencies are “key knowledge, skills, abilities, and attitudes that the health workforce should possess to effectively deliver essential public health functions.” [16] Indian professional education councils for nurses, midwives, and medical doctors define competencies expected from graduates [17,18]. These competencies mostly focus on knowledge but are not rooted in PHC practice, particularly in the public sector, resulting in a knowledge-practice gap [19]. Other attempts at defining health worker competencies in India include those for mid-level managerial and supervisory public health professionals in Uttar Pradesh, for pharmacists, and for medical laboratory technologists [16,20,21]. Each of these studies attempted to understand the competencies of a specific cadre.

To successfully implement a team-based approach, there is a need to identify the collective competencies of the PHC team. This paper presents findings from a study that identified key PHC team functions and corresponding competencies. To our knowledge, this study is the first of its kind in the country to identify PHC team competencies using a bottom-up approach. Study findings can inform the design and rollout of competency-based capacity-building efforts; it can also guide wider PHC workforce recruitment and retention policies.

This study was conducted in the district of Bhavnagar, Gujarat. Bhavnagar is a coastal district in Gujarat state with a population of about 2.6 million. The district is divided administratively into ten blocks (Talukas) with 48 tier-1 Primary Health Care facilities, catering to 30,000 population, and 251 sub-health center level facilities under them, each catering to 5,000 population. The majority of the sub-health entry-level facilities have been upgraded to AAMs. This district health system is managed by the Chief District Health Officer.

**Box 1. India Primary Healthcare Support Initiative.**

The India Primary Healthcare Support Initiative (IPSI) is a consortium of public health schools and organizations in India, led by the Johns Hopkins Bloomberg School of Public Health, Baltimore and funded by the Gates Foundation. The overarching goal of IPSI is to support the national, state and district health authorities in strengthening primary health care (PHC). In Gujarat, the state consortium partners are the Indian Institute of Public Health, Gandhinagar and State Health System Resource Center with support from the district health office of Bhavnagar. In Bhavnagar, IPSI activities focus on (1) improving quality of care (2) supporting facility service readiness and (3) capacity building. At the time of this study, IPSI was piloting an intervention to strengthen AAM team functioning in Bhavnagar using a mentoring approach.

## Methods

Ethics statement: This study was approved by institutional ethics review boards of Johns Hopkins Bloomberg School of Public Health (21610/MOD4076) and Indian Institute of Public Health, Gandhinagar (DMIHER (DU)/IEC/2023/52). Informed oral consent was obtained from participants; in line with the ethics approvals, it was not mandatory to document or have witnesses present for the same. There was no deviation from the approved study protocol. Additional information regarding the ethical, cultural, and scientific considerations specific to inclusivity in global research is included separately.

Targeted literature review: We conducted a targeted search of peer-reviewed and grey literature for evidence on PHC team cadres, their functions, and competencies. The PHC team in this study comprised the cadres at AAMs in Bhavnagar. We reviewed Indian policy documents to ensure alignment with existing PHC context and priorities including guidelines and standards such as Indian Public Health Standards (IPHS), 2022 for AAMs, the Ayushman Bharat-Health and Wellness Center operational guidelines, and the country’s National Health Policy, 2017 [10,22,23].

Consultations with experts and PHC team members: To ground the list of PHC team functions in Bhavnagar’s context, two sets of consultations were held. The first was held with technical experts (hereafter referred to as “expert consultation”) and the second with PHC teams (hereafter referred to as “PHC team consultations”). Consultations were facilitated by IPSI research team members with a master’s or PhD degree in public health and familiarity with the Indian health care system. In the expert consultation, a two-day voluntary workshop was held in April 2024 in Bhavnagar with a diverse group of experts, including faculty from public health institutions, nursing and medical schools, district health officials, and public health specialists. In the first round of group work, participants reviewed and edited an initial list of PHC team functions derived from policy documents. These were compiled across groups to obtain an exhaustive list of PHC team functions. In the second group work exercise, participants listed knowledge, skills, and attitudes that the PHC team needs to possess to perform each function effectively. Results from the group work were organized as competencies and presented back to the participants for validation on day two. PHC team consultations were held with members of three purposively selected PHC teams in Bhavnagar in May 2024. Participant recruitment occurred in April and May 2024. The teams were identified in consultation with the district health authorities in Bhavnagar. Consultations were held at the AAM facilities; at each facility, the new community health officer cadre was consulted individually while all other cadres were consulted as a group. In each consultation, participants reviewed and edited the list of functions, discussed their perceptions of working as a team, and using the list of competencies developed during the expert consultation, prioritized and identified gaps in the list. No formal consensus building methods (such as Delphi) were used during the consultations.

Data analysis: To balance rigor with efficiency, group work in the expert consultation utilized standardized templates to gather information on knowledge, skills and attitude (see S2 Table). The data (i.e., knowledge, skills and attitude) were further organized in an excel based analytical matrix that enabled easy familiarization with the data to allow reduction and its re-arrangement based on emerging thematic patterns. These themes were labeled inductively and distilled into overarching competency domains. Only excel was used to facilitate analysis; no analytical software was used. The analysis was predominantly conducted by two IPSI research team members with a Masters or Doctoral degree in Public Health and deep knowledge of the Indian healthcare system. The emerging findings were discussed with peers within the IPSI team. The compiled PHC team functions and competencies were then presented back to the experts on day two of the workshop to elicit feedback, which was noted and incorporated in the findings. The traditional analytical steps of transcribing, coding and saturation were skipped to enable rapid incorporation of findings into an intervention design (see Box 1). During the PHC team consultations, the list of PHC team functions and competencies was presented to elicit their edits and feedback, which were incorporated in the final list. Credibility of study findings was strengthened through respondent validation during the expert consultation, triangulation of findings across experts and PHC team members and documenting the detailed process.

## Results

This section first presents literature review findings which includes results on the PHC team composition relevant to Bhavnagar’s context and the preliminary list of PHC team functions. It then presents results from the expert and PHC team consultations, discussing the list of PHC team functions and competencies identified through consensus.

PHC team composition: PHC teams in Bhavnagar comprise a nurse or Ayurveda doctor, known as a mid-level health provider or community health officer (“CHO”), a male and a female multi-purpose worker (“MPW-M” and “MPW-F”) with the MPW-F also known as an Auxiliary Nurse Midwife or Female Health Worker, and community health workers known as Accredited Social Health Activists (“ASHA”) (see Table 1).

**Table 1:** Select characteristics of PHC team cadres*.

	Community Health Officer (CHO) or Mid-level Health Provider (MLHP)	Multi-purpose Worker, Male (MPW-M)	Multi-Purpose Worker, Female (MPW-F), Auxiliary Nurse Midwife (ANM), or Female Health Worker (FHW)	Community health worker: Accredited Social Health Activist (ASHA)
**Year of cadre creation**	2018 [24]	1974 [25]	1950s [26]	2005 [27]
**Educational qualifications**	BSc** or GNM*** in Community Health/Nursing or Ayurveda practitioner from a recognized institution. [10]	Minimum grade 12 graduate in science or biology. Skilled at the paramedic level, specifically able to provide laboratory, pharmacy, and counselling care. [25]	Two-year, certified Diploma in Auxiliary Nursing and Midwifery from an institute recognized by the Indian Nursing Council. [26]	Grade 10 or higher. [27]
**Required Training**	6 months pre-service certificate course in Community Health, 3 days pre-service training on telehealth use. 5–7 days annual ongoing/ supplementary/ refresher training. [10]	10 days pre-service training on screening and prevention of non-communicable diseases, and use of digital information platforms. 3–5 days annual ongoing/ supplementary/ refresher training. [10]	Same as MPW-M, with an additional 26 days of pre-service training for skilled birth attendants and reproductive care skills. [10]	8 days pre-service training, 25 days skill-based, programmatic training. 15 days annual ongoing/ refresher/ supplementary training. [10]
**Roles**	1. Clinical functions including outpatient department services, 2. Public health functions for prevention, promotion, and disease surveillance, 3. Oversee efficient management of the health facility. [10]	1. Community identification of Malaria, Leprosy, and Tuberculosis 2. Preventive health care including school health and nutrition 3. Family planning services. [10]	Facility and community-based RMNCHA^$^ services including immunization. [10]	1. First-contact care at the community level. 2. Prevention, promotion in the community 3. Screening 4. Surveillance [10]
**Supervisory Pathways**	Reports to the Medical Officer at the Primary Health Centre^&^. [28]	Reports to the Medical Officer at the Primary Health Centre. Since 2018, is meant to report to CHO. [25]	Previously reported to Lady Health Visitor, a more senior and experienced MPW-F, who further reported up to the Medical Officer at Primary Health Center. Since 2018, is meant to report to CHO. [26]	Reports to supervisory cadre of ASHA Facilitator. Also reports to the MPW-F at the facility and Anganwadi worker (under the Ministry of Women and Child Development). Is also accountable to community-based committees. [13]
**Type of Employment**	Contractual government employee. [10]	Salaried, government employee. [27]	Salaried, government employee. [27]	Voluntary, selected by community and/or by MPW-F. [27]
**Payment Mechanisms**	Salaried with a fixed monthly component and a variable monthly component tied to team performance. [10]	Fixed monthly salary. Recent addition of team-based incentive, on top of the salary. [10,27]	Fixed monthly salary. Recent addition of team-based incentive, on top of the salary. [10,27]	Fixed monthly incentive, with additional performance-based incentives for services provided. [27]

* can vary across states

** Bachelor of Science in Nursing (B.Sc.)

*** General Nursing and Midwifery (GNM)

$Reproductive, Maternal, Newborn, Child, and Adolescent Health (RMNCHA)

&Primary Health Centers are facilities that are one level over the subhealth center AAMs in the public health sector.

A key component of India’s reform to strengthen public sector PHC was the introduction of the CHO cadre at AAMs in 2018 (see Table 1) [22]. Before that, care was delivered by MPWs and ASHAs. The CHO is responsible for providing clinical services through outpatient care, preventive, and promotive health services, disease surveillance, and facility management [10]. The MPW-F provides the bulk of reproductive, maternal, child, and adolescent health services (RMNCHA) including immunization [10]. The MPW-M conducts community identification of malaria, leprosy, and tuberculosis, provides preventive health services at schools, and renders family planning services to men [10,22,25]. ASHAs are a community-based, voluntary cadre, envisioned to deliver health services to the last mile; they track and mobilize communities to access health services, conduct awareness and prevention activities through health days and household visits, and support the delivery of community-based services for RMNCHA and NCDs [10,22,27]. The employment type, reimbursement methods, and supervisory pathways vary across the cadres (see Table 1).

PHC team functions: Based on the literature review, an initial list of PHC team functions was created which included taskwork processes, i.e., activities linked to the team’s objectives, and teamwork processes, i.e., the way team members work together on interdependent tasks (see S1 Table). Thus, functions were not separated by cadre-wise responsibilities but focused on their work as a unit. Functions were divided into facility and community levels and incorporated personal, primary care services, public health functions, and administrative tasks. At the community level, functions focused on health communication and outreach, implementation, and monitoring. At the facility level, functions included screening, treatment, and management of health conditions and facility management such as maintaining inventory and records. This list of PHC team functions was discussed during the expert and PHC team consultations.

The expert consultation was held with a total of 35 participants (see Table 2). Participants included faculty from local government nursing and medical colleges (51%), experts from national and international academic institutes (34%), government health administrators (11%), and public health specialists (3%). Of the 35 participants, 51% were from within Bhavnagar district. PHC team consultations were held with three AAM teams with representation from all team member cadres.

**Table 2:** Participant characteristics at expert consultation.

Demographic characteristics of expert consultation participants (N = 35)	
***Sex***	Number (%)
Male	15 (43%)
***Professional Location***	
Within the Bhavnagar district, Gujarat	18 (51%)
***Type of Institution/ Organization***	
National, and international academic institutes	12 (34%)
Government Medical College	7 (20%)
Government Health Administration	4 (11%)
Government Nursing College	11 (31%)
Public health specialist	1 (3%)
	35 (100%)

The expert and PHC team consultations resulted in changes to the list of PHC team functions. For example, policy guidelines envision AAMs to address gender-based violence (GBV) and facilitate patient support groups (see S1 Table) [22]. However, both experts and PHC teams excluded these functions from the list. Additionally, managing absenteeism at the AAM was not seen as the PHC team’s responsibility and was excluded. The use of population-based analytics to plan services was revised to incorporate a broader scope of reviewing, using, and interpreting data to plan services and improve service quality. Both experts and PHC teams felt that teams in Bhavnagar usually do not respond to disease outbreaks but cited the example of the COVID-19 pandemic as an exception, due to which the function was retained. The consultations also resulted in the addition of a few PHC team functions at the community level; these were (i) knowing the community and its health priorities, (ii) recording and reporting health events in portals, (iii) conducting meetings, (iv) building rapport with communities and (v) ensuring treatment adherence among high-risk patients. At the facility level, the following PHC team functions were added: (i) handling cases of resistance/ service refusals and treatment dropouts, (ii) managing patient grievances, (iii) using digital communication, and (iv) undertaking quality improvement processes and certification. PHC teams additionally felt that they were often involved in enrolling community members into government benefit schemes.

**Fig 1:**
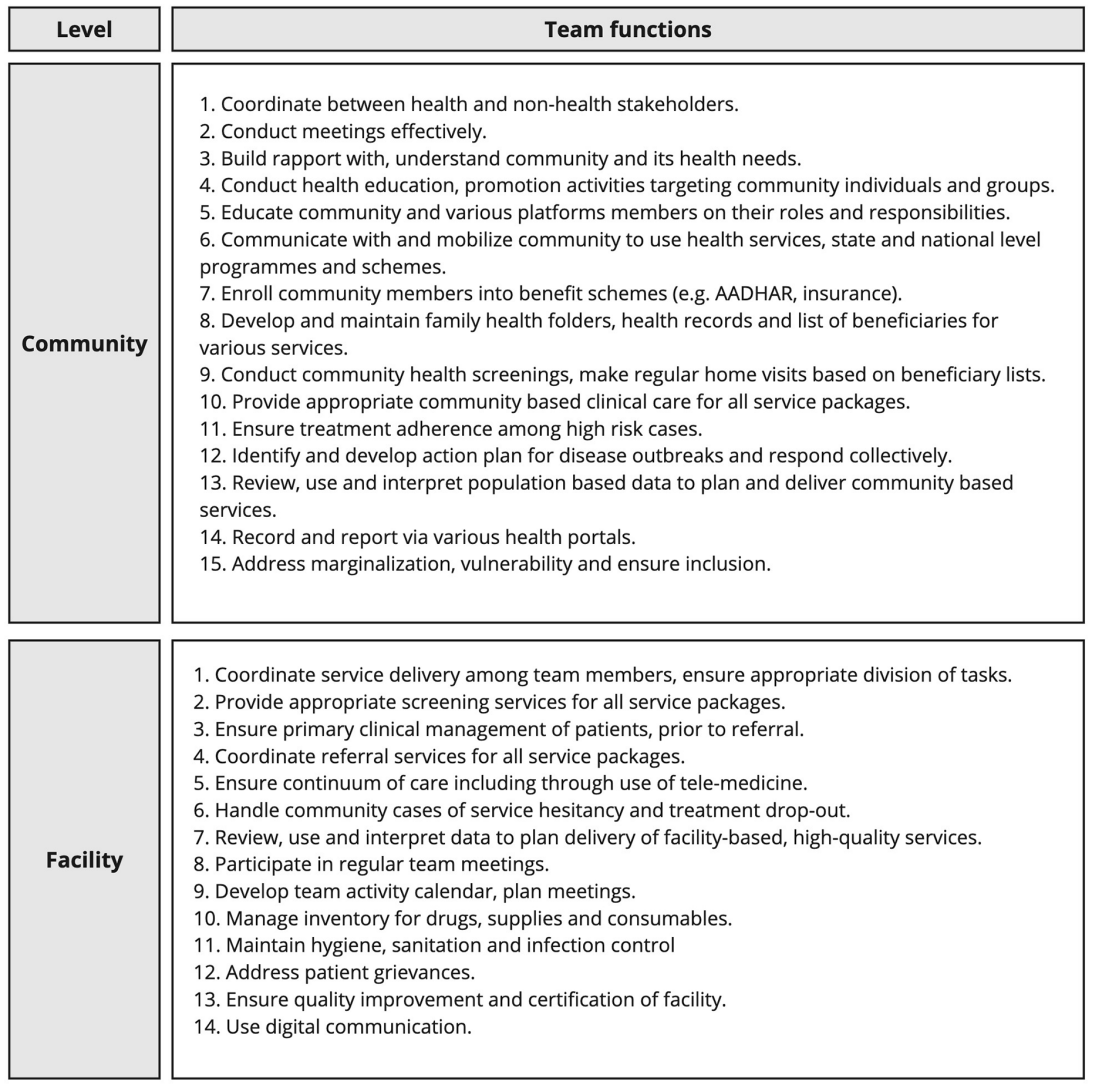
Summary functions of the primary health care (PHC) team.

PHC team members perceived team functions through a cadre-specific lens, providing examples of the role of each cadre as opposed to combined roles of the team. Additionally, cadre-wise functions identified by teams were highly verticalized, particularly for the MPW-M and MPW-F. This is aligned with their roles described in policy documents and the literature (see Table 1). Further, perceptions towards the CHO’s role varied; while CHOs viewed themselves as accountable for all services rendered at the facility, other cadres believed CHOs should only be responsible for delivering “newer” service packages while performing a supportive role in the historically provided RMNCHA and communicable disease services. The final list of PHC team functions in Bhavnagar is presented in Fig 1.

**Fig 2:**
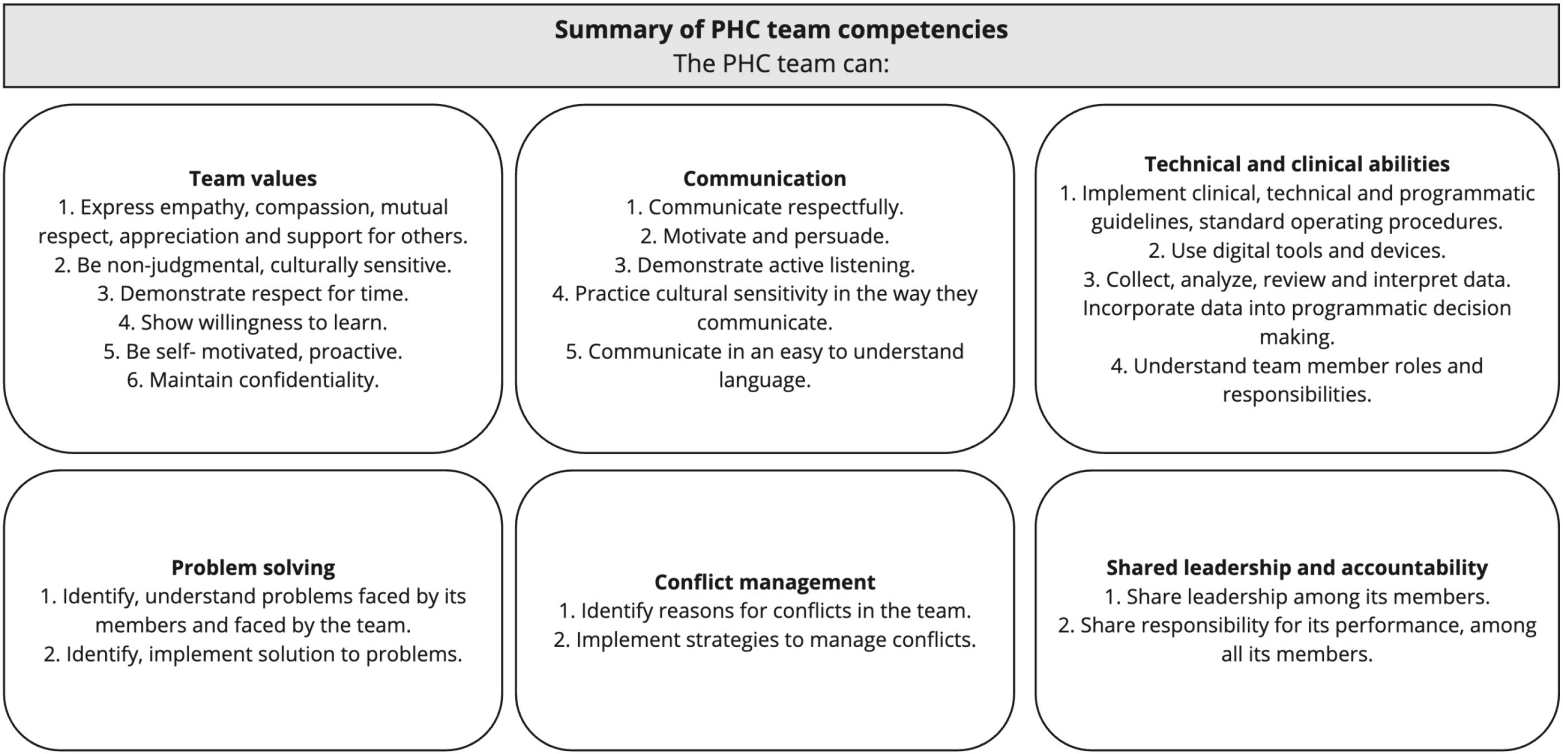
Primary health care (PHC) team competency themes.

PHC team competencies: PHC team competencies based on expert and PHC team consultations were divided into six themes: technical and clinical, communication, shared leadership and accountability, problem-solving, conflict management, and team values (see Fig 2). Overall findings against each competency theme are discussed below.

Technical and clinical competencies: A broad theme of technical and clinical competencies included those on the use of digital tools; and the collection and analysis of routine data and its use in programmatic decision-making. The PHC teams felt that collection and analysis, review, and interpretation of routine data would help in understanding the community health profile and planning service delivery. This theme also included understanding the roles and responsibilities of all team member cadres; and knowledge of and ability to implement clinical, technical, and programmatic guidelines and standard operating procedures (SOPs). While clinical skills are taught during cadre-specific state, and district government-led trainings, the need for a more hands-on, practical approach was identified as a way to fill gaps in the basic clinical competencies of all team members and enhance their confidence to render these clinical services.

*‘So far, we have been taught counselling etc. theoretically, but practically not much. Like we know we must fill out registers, and we must get signed, but we don’t really know how to do it. We want to learn all this physically and not through video.’: PHC team participant*

Communication: Competencies pertaining to communication, both among team members and with the community, were prioritized in all consultations. These included knowledge and ability to communicate respectfully, in a manner that is motivating, culturally sensitive, and incorporates active listening.

Shared leadership and accountability: Shared and equal leadership within the PHC team emerged as an important competency from both rounds of consultations. Broadly, all members of the PHC team considered the Medical Officer at the Primary Health Centre as their leader. Within the PHC team though, members held different views; CHOs identified themselves as leaders of the PHC team which was aligned with the training they received. However, other team members showed little willingness to accept the CHO as their team leader. Shared accountability was also considered critical, wherein each team member takes collective responsibility for the team’s performance.

*‘For 6 months we have been told during training that we are the leaders….and you have to check the record, but this is not been possible….everyone’s responsibilities have changed and shifted, but they have not been told about it.’: CHO, PHC team participant*

*‘Everyone is a leader. ASHA is a leader too. Only when she goes to the field and finds us people* *can we work. We can’t be everywhere. Sab ek samaan.* (Everyone is equal)’: *PHC team participant*

Problem-solving: This theme included knowledge of and ability to identify challenges faced by individual team members and by the team overall. It also included the ability to identify root causes of the challenges and identify and implement solutions to address the challenges. The consultations with the PHC teams highlighted the importance of a health system environment that fosters problem-solving among teams as opposed to fault-finding by supervisors.

*‘There is a hierarchical system here. Everyone finds fault with us- tells us you have not achieved this...see, this indicator less than 50% for example, they will tell you. But no one tries to ask us why it is low.*’: *PHC team participant*

Conflict management: Knowledge of and ability to identify reasons for conflicts and implement strategies to manage them were considered another key competency. This was perceived as integral in the context of conflicts arising within the team since the introduction of the new CHO cadre.

*‘For so many years ASHA, FHW…. We have been working together. But since this CHO post has come, then everywhere there are fights…, We feel there is a need for something for common understanding between the cadre.’: PHC team participant*

Team values: The ability of team members to express certain subjective feelings and attitudes in their behavior was considered another key competency. Participants in the expert consultation identified these attitudes and feelings as empathy, compassion, a non-judgmental demeanor, mutual respect, respect for another’s time, self-motivation, and a proactive outlook. The PHC teams identified confidentiality, a need to support and learn from one another, and positive thinking as some of the team values.

## Discussion

This study identified PHC team functions and competencies in Bhavnagar district, Gujarat. To our knowledge, this is the first attempt to identify competencies of the PHC team as a unit in India. This study is timely given the policy inclination to reorient PHC towards a comprehensive, team-based approach [10]. Additionally, through a bottom-up process and the involvement of a diverse set of key stakeholders, this study aligns itself very closely with practice and program implementation.

PHC team functions spanned the community and facility levels and included taskwork processes that directly help the team achieve its objectives and teamwork processes that enable team members to align their interdependent tasks. The collective competencies that the PHC team must possess to effectively perform these functions include technical and clinical competencies, communication, shared leadership and accountability, problem-solving, conflict management, and a set of team values. While the global literature on PHC *team* competencies is scant, evidence from a scoping review for delivering integrated care and another from a Chilean study identified similar competencies that PHC team members should possess; besides technical skills such as conducting home visits and counseling patients, these studies included shared decision making, problem solving, communication, collaboration, leadership and teamwork [29,30]. The Chilean study diverged from this study in that it was geared towards identifying cadre-wise competencies of each individual in the PHC team, while this study focused on identifying core competencies that every PHC team member should possess [30]. Similar to our study, global frameworks for identifying *individual* health worker competencies generally present brief competency statement(s) under a domain [31,32]. Some frameworks further break down the competencies into behaviors that demonstrate associated knowledge, skills and attitudes and/or sub-competencies based on level of expertise, which our study did not aim to do. We do observe some alignment in competency domains and themes between these frameworks and our findings, e.g., communication, leadership, conflict management or collaboration and personal conduct.

This study’s findings have an important implication for designing PHC team capacity-building strategies; to facilitate strengthening of core competencies among all PHC team members, cadre-specific technical or programmatic trainings must be supplemented with team trainings that enable all team members to perform teamwork processes [33]. The literature identified that team trainings can take the form of classroom-style or didactic trainings, simulation exercises, workshop-style group exercises, or on-site team reviews. Evidence shows that training team members together facilitates better teamwork [34,35]. Moreover, team trainings that incorporate team-building exercises can support PHC teams towards emergent states of trust, respect, and compassion [34]. The feasibility and effectiveness of such team trainings should be explored.

While technical and clinical competencies were considered important, the need to strengthen “softer” competencies among PHC team members emerged as a priority. Evidence from existing frameworks on team dynamics in the literature supports our findings; one framework analyzing the role of team dynamics in primary care team effectiveness identified the shared understanding of team goals and roles, accountability, decision-making, conflict resolution, and communication as key mechanisms that influence team behavior and effectiveness [36]. Similarly, frameworks on relational coordination depict its association with better task quality and efficiency; relational coordination includes interactions driven by shared goals, shared knowledge, mutual respect, and the nature of communication, i.e., its frequency, timeliness, accuracy, and problem-solving orientation [37]. Studies also report an association of team climate with patient experience and quality of clinical care wherein team climate encompasses attitudes and behaviors of team members for communication and decision-making, shared goals and responsibilities, commitment to excellence, and exchange of knowledge and practices [38,39]. Our finding implies that PHC team trainings and other capacity-building efforts must incorporate “softer” competencies beyond clinical and technical competencies.

Finally, we identified a need to create a policy and organizational environment that supports team-based care. There was a lack of coherence among PHC team members regarding team membership and leadership. India’s PHC policy lays the foundation by initiating the move towards team-based care [10]. However, evidence shows that this reorganization of the team must be coupled with supporting interventions such as payment policies and reward systems, defined roles, responsibilities and tasks, and hiring and training protocols, among others [36,37,40,41]. Studies have also identified within-team hierarchies as barriers to team-based care [42]. Our findings point to a need to address challenges likely associated with the staggered introduction of PHC team member cadres, historical verticalization of their roles, variable reimbursement and supervision mechanisms, and power dynamics within teams (see Table 1).

In summary, our findings point to three main implications for the implementation of team-based PHC. The first pertains to the *modality* of PHC team capacity building and suggests PHC team trainings to supplement existing, cadre-specific trainings. The second pertains to the *content* of capacity-building efforts and recommends addressing the identified competencies with greater emphasis on those relating to communication, shared leadership and accountability, problem-solving, conflict management, and team values. The third pertains to the broader health system environment that must support the routine practice of team-based care.

Our study has a few limitations. We identified core competency themes for a specific type of PHC team in Bhavnagar district. This was aligned with the priorities of the district health authority in Bhavnagar. We anticipate that these competencies will vary based on team composition and its functions. We also do not identify detailed competency statements/ behaviors/ expertise levels. The identification of competency themes was meant to inform and guide interactions between mentors and mentee AAM teams as part of the IPSI intervention in Bhavnagar (see Box 1). Since the study was limited to understanding PHC team competencies in one district, the findings should be validated in other districts and states prior to application. Further, validation of the PHC team functions and competencies was done through group consultations with PHC team members. Since these consultations involved cross cadre members of the team, hierarchies among team members may have restricted responses from some. This study also did not aim to identify ways to measure PHC team competencies. The absence of such a measurement tool makes it challenging to comment on the measurability of the competency domains our study has identified. Future research should focus on developing and validating competency statements for PHC teams more broadly, the measurement of competencies among PHC team members, and the study of interventions to enhance team competencies.

## Conclusion

To reorient PHC towards a team-based approach, capacity-building efforts need to address competencies that the PHC team possesses as a unit. Our study findings indicate that these team competencies include the more traditional technical and clinical competencies, as well as, competencies that strengthen ways in which teams work together. The latter include competencies around communication, shared leadership, problem-solving, conflict management, and team values. Additionally, these competencies should be strengthened by training team members together. Team-based approaches to service delivery also need to be supported by policy and organizational interventions including payments and reward systems, clearly defined roles and responsibilities, and supervisory pathways.

## Supporting information

S1 Table Initial list of PHC team functions for consultations. (DOCX)

S2 Table PHC team functions and corresponding knowledge, skills, attitudes as per expert consultation. (DOCX)

S1 Checklist Inclusivity in global research checklist. (DOCX)
