# Supplementary material for: Identifying competencies of primary health care teams in Bhavnagar district, Gujarat
Source: PLOS Glob Public Health. 2026 Jul 27;6(7):e0006861. doi: 10.1371/journal.pgph.0006861 (PMC13405124; doi:10.1371/journal.pgph.0006861)
Supplement: S1 Table — (DOCX) [file pgph.0006861.s001.docx]

S1 Table

Initial list of PHC team functions for consultations

| **Level** | **PHC team functions**  **PHC team members need to….** |
| --- | --- |
| Community | Coordinate across different health and non-health stakeholders  (e.g., coordinate with AWW to conduct VHNDs, coordinate with JAS members to conduct meetings, coordinate with PHC and block-level staff to respond to disease outbreaks, etc.) |
|  | Conduct health education and promotion activities  (e.g., counsel tobacco users to quit tobacco, conduct yoga sessions for the community, support formation and handholding of diabetes patient support groups, educate households to prevent mosquito breeding, etc.) |
|  | Mobilize the community by communicating HWC services to the community  (e.g., inform community members on availability of service packages, diagnostics, timings of HWC facility, etc.) |
|  | Two-way communication of information on disease outbreaks  (e.g., communicate to block/district on the rise in symptoms of a disease in the community, communicate preventive measures for COVID-19 to the community) |
|  | Develop and maintain family health folders, individual health records, and vital statistics. |
|  | Identify and maintain a list of beneficiaries for various services like eligible couples for contraceptives, pregnant women, hypertensives, etc. |
|  | Conduct health screenings in the community  (e.g., completion of CBAC forms, NCD screening, antenatal care, screening camps for eye care, etc.) |
|  | Using population-based analytics to plan for community-based services  (e.g., plan home visits for HBNC, palliative care, TB treatment based on a list of beneficiaries, etc.) |
|  | Visit homes in the community based on beneficiary lists  (e.g., make home visits for HBNC, palliative care, TB treatment, etc.) |
|  | Provide appropriate community-based clinical care for 12 service packages  (e.g., HBNC, palliative care, etc.) |
|  | Respond to disease outbreaks  (e.g., coordinate testing and result reporting for COVID-19) |
|  | Address issues of marginalization and violence against women |
| Facility | Provide appropriate screening services for all service packages |
|  | Provide appropriate patient assessment services for all service packages |
|  | Provide appropriate disease management services for all service packages |
|  | Provide appropriate referral services for all service packages.  e.g., coordinate referrals to and from other facilities and community |
|  | Coordinate service delivery with team members  e.g., coordinate case management, patient workflow, task division, screening days, special day health clinics, etc. |
|  | Participate in regular team meetings |
|  | Manage absenteeism within the team |
|  | Manage untied funds |
|  | Manage inventory for drugs, supplies, consumables |
|  | Maintain hygiene, cleanliness, infection control |
|  | Maintain records (paper and/or digital) for service delivery, inventory management, finances, reporting |
