# Supplementary material for: Identifying competencies of primary health care teams in Bhavnagar district, Gujarat
Source: PLOS Glob Public Health. 2026 Jul 27;6(7):e0006861. doi: 10.1371/journal.pgph.0006861 (PMC13405124; doi:10.1371/journal.pgph.0006861)
Supplement: S2 Table — (DOCX) [file pgph.0006861.s002.docx]

S2 Table

PHC team functions and corresponding knowledge, skills, attitudes as per expert consultation

| **Level:** | **PHC team functions.** PHC team members need to: | **Knowledge**. PHC team members must have knowledge of: | **Skills**. PHC team members must be skilled in: | **Attitude**. PHC team members must be able to: |
| --- | --- | --- | --- | --- |
| Community | Coordinate across different health and non-health stakeholders. | Guidelines and functioning of various committees/sectors | Communication, coordination, conflict management, and leadership. | Problem solve and  actively listen. |
|  | Understand the community and its health priorities. | Basic demographic profile, geographical distribution, common endemic diseases, hereditary disorders, and socio-cultural practices. | Communication,  leadership, trust building, data analysis and collection, use of information technology platforms, and record keeping. | Maintain confidentiality and accountability, collaborate across teams, and use non-discriminatory behavior. |
|  | Undertake collective team planning to deliver health education and promotion activities, at individual/ peer group level. | Health education principles, community culture, and audio-visual aids. | Local languages, working with team members, and communication with community individuals and groups. | Participate, listen, and demonstrate efficiency through teamwork. |
|  | Mobilize the community as a team by communicating PHC services, including state and national programs. | Benefits of services to the community, and the appropriate clinical skills to deliver services. | Communicating the importance of services to the community, in their preferred language. Understanding communities’ willingness, traditions, norms, customs, and cultural/ spiritual practices. | Respect community beliefs and be humble. |
|  | Develop and maintain health records. | Program guidelines for data management, records keeping, and calculation of rates/proportions. Use of digital tools, and demographic profile of the catchment population of the community. | Data analytics, digital literacy, problem-solving, advocacy, and judging data quality. | Problem-solving, collaborate within teams, and take  responsibility. |
|  | Identify and maintain a list of beneficiaries for various services. | Guidelines and latest reporting formats, and clarity of terms of reference/job functions. | Data collection and analysis, and use of information technology systems. | Motivate others and be culturally sensitive. |
|  | Conduct health screenings in the community. | Screening guidelines and referral system. | Community mobilization and planning, communication, use of relevant tools and IT platforms, and building rapport. | Lead and participate, contribute to team motivation. |
|  | Review, use, and interpret population-based data to plan for community-based services, to improve service quality. | The indicators and purpose of data, including knowledge of the population and their needs. | Data analytics, and data-based decision making. | Demonstrate empathy and understand the importance of data. |
|  | Regularly visit homes in the community based on beneficiary lists. | The physical assessment and health services protocols. | Conducting physical assessment of beneficiaries. | Show humility, respect community beliefs, and demonstrate an interest in new knowledge. |
|  | Provide appropriate community-based clinical care for all service packages. | Clinical and referral pathways, the health profile of the catchment population, specific program guidelines, and the understanding of community profile including taboos and customs. | Communication, planning of services, persuading community members, and delivering clinical services. | Respect community and team members and demonstrate responsibility and interest in learning from others. |
|  | Address issues of marginalization/ vulnerability and ensure inclusion. | Concepts of marginalization and vulnerability. | Communicating with vulnerable populations, and coordinating with non-health sectors. | Exercise non-judgment. |
|  | Record and report various health events in portals. | Portals, reporting systems, timelines, and information for technology use. | Operating portals, dashboards, and information technology systems. | Demonstrate timeliness, accuracy, and confidentiality, as well as sincerity and credibility with data. |
|  | Conduct meetings effectively. | Meeting agenda. | Managing meetings, including conducting, organizing, and documenting meetings. | Demonstrate timeliness and appreciation towards others. |
|  | Educate community and platform members on their roles and responsibilities. | Community platform and its roles and responsibilities. | Motivating, recognizing, and appreciating community members. | Show passion and motivation for community engagement. |
|  | Identify and develop action plans for disease outbreaks and respond collectively. | Disease surveillance, understanding of health system, and epidemic investigation. | Coordinating with the team members, interpreting data, leadership, communication, and technical management of disease outbreaks. | Take initiative, be empathetic, and show the presence of mind. |
|  | Build rapport with the community. | The community and its culture. | Communication. | Actively listen, with a nonbiased approach. |
|  | Ensure adherence to high-risk case treatment. | High-risk cases and referral systems, and where to access real-time data. | Active surveillance and follow-up, assessment of high-risk cases, primary clinical management of cases, stock management (regular indenting), and data interpretation. | Demonstrate diligence, collaboration, accountability, and promptness, when following up for referrals. |
| Facility | Handle community cases of service use hesitancy/ treatment drop-outs. | The reasons for resistance/ hesitancy, community customs, taboos, and stigmas. | Root cause analysis. | Show a positive attitude. |
|  | Provide appropriate screening services for all service packages. | All screening services, protocols, and reporting formats. | Counselling and referral management, managing logistics, and communication. | Use compassion and active listening with patients. |
|  | Coordinate appropriate referral services for all service packages. | The standard operating procedures for referrals, and clinical management (diagnosis, history, examination). | Clinical examination (diagnostics, symptoms, history taking), and communication between team members. | Provide guidance and recognize/ appreciate team members. |
|  | Coordinate service delivery with team members. | The roles and responsibilities of team members, including an understanding of program guidelines. | Conflict management, communication, and leadership. | Collaborate with team members. |
|  | Review, use, and interpret facility-based data to plan for quality facility services. | Data methods and understanding of data quality and use. | Data analysis, interpretation, and use in decision-making.  Documentation, use of information technology systems, and critical thinking. |  |
|  | Participate in regular team meetings/ huddles. | The work is undertaken by each team member, including the information on each meeting topic on the agenda. | Meeting management, leadership, team coordination, active listening, problem-solving, and time and conflict management. | Maintain a regular routine, provide constructive feedback, and show motivation and active participation. |
|  | Develop activity calendar and plan meetings. | The standard operating procedures of services and planning are based on experience and data. | Microplanning. | Take initiative. |
|  | Manage inventory for drugs, supplies, and consumables. | Inventory management, appropriate indenting, and use of digital tools. | Inventory management, and data use and interpretation. | Be organized. |
|  | Maintain hygiene, cleanliness, and infection control. | Protocols for facility maintenance, infectious diseases, and importance of hygiene.  Infection control guidelines and practices. | Leadership, implementing infection control guidelines, and organization. | Show respect and pride in their facilities |
|  | Manage grievances and patient satisfaction. | Common patient complaints and redressal mechanisms. | Problem-solving and managing challenges successfully within a team. | Take initiative and show compassion for patients. |
|  | Ensure task division among team members. | The job chart and staff qualities including  strengths and weaknesses. | Supervision and delegation of tasks, team review, and evaluation. | Act with honesty, motivation, and in a cooperative manner on a team, and extend support and constructive feedback. |
|  | Communicate digitally. | Information technologies. | Using digital gadgets, information technology platforms, and dashboards. | Use technology with ease. |
|  | Ensure primary management of patients at AAM before referral. | Updated standard operating procedures and clinical protocols. | Clinical management. | Demonstrate a constant interest in learning more. |
|  | Ensure quality improvement and certification at the facility level. | Concepts of quality. Understanding of National Quality Assurance Standards and processes involved. | Leadership, observation, and documentation. | Show motivation and self-reflection. |
|  | Maintain a continuum of care including through telemedicine. | Information technology system and referral systems. | Use of information technology systems and remote system operations. | Listen, and show patience, initiative, and assertiveness. |
